# Supplementary material for: Evaluating large language models on medical evidence summarization
Source: NPJ Digit Med. 2023 Aug 24;6:158. doi: 10.1038/s41746-023-00896-7 (PMC10449915; doi:10.1038/s41746-023-00896-7)
Supplement: Supplementary file 1 — SUPPLEMENTAL MATERIAL [file 41746_2023_896_MOESM1_ESM.pdf]

Supplementary materials

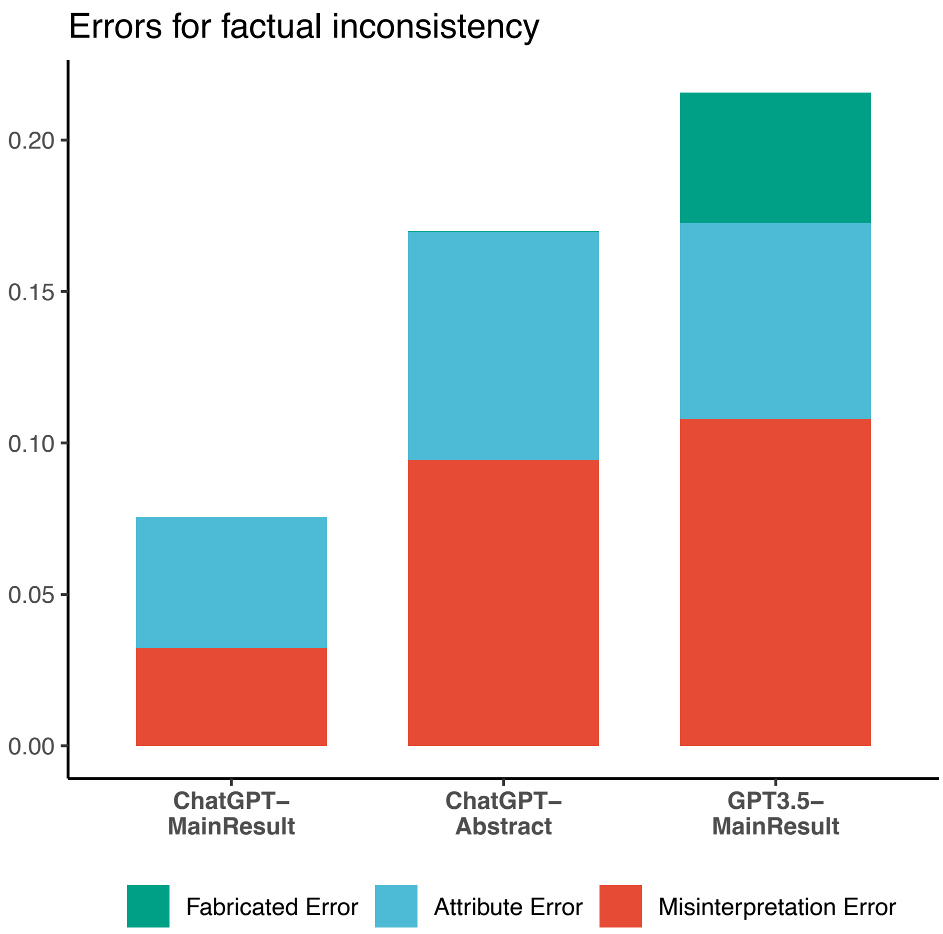

Supplementary Figure 1. Statistics of errors for factual inconsistency across models.

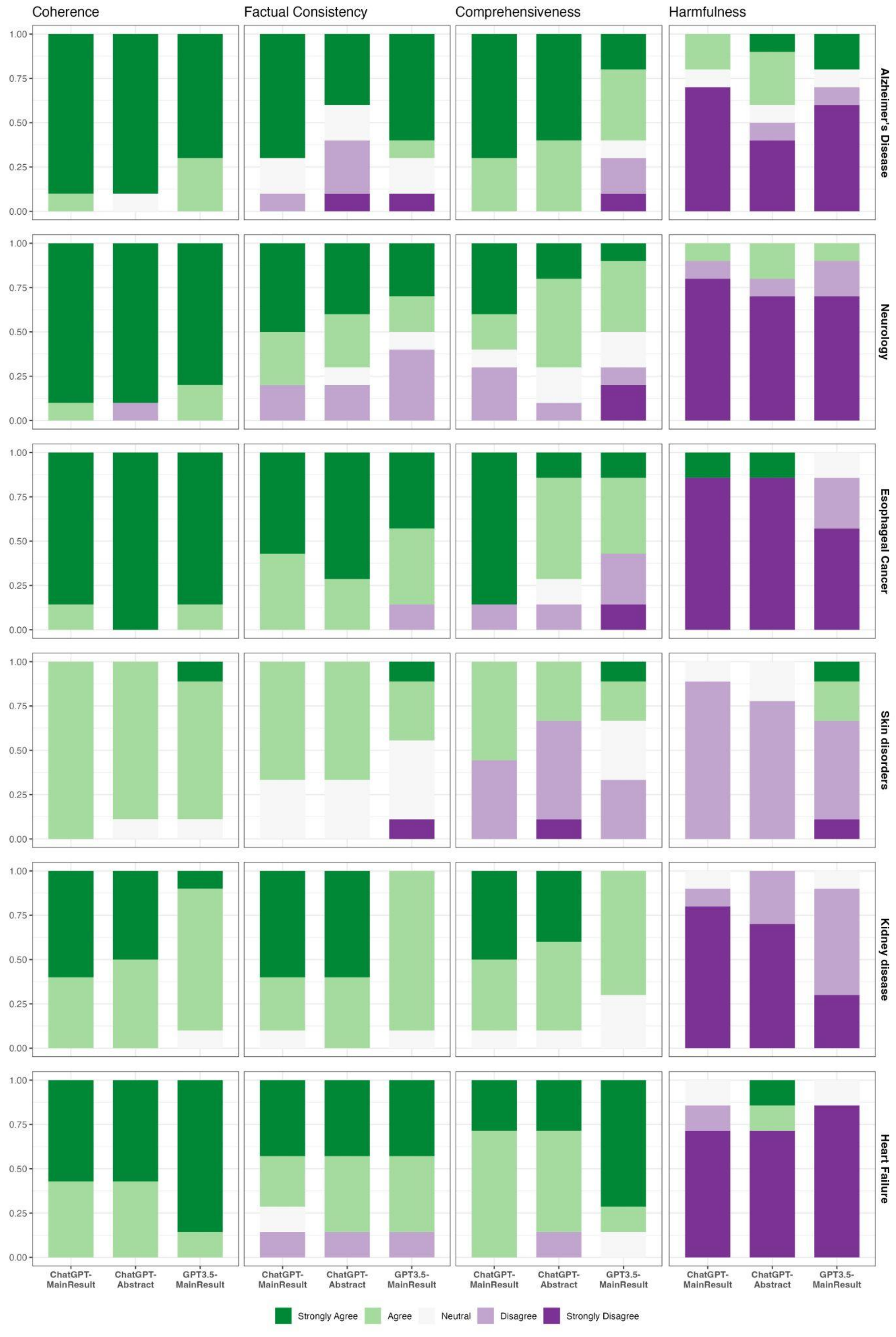

Supplementary Figure 2. Annotator vote distribution of summaries generated by different summarization systems in 6 clinical domains.

Supplementary Table 1. The average number of words in the generated summarization

Domain	Gold standard summary	ChatGPT-MainResults	ChatGPT-Abstract	ChatGPT3.5-MainResult
Alzheimer's disease	114	105	111	93
Kidney disease	95	117	123	96
Neurological conditions	101	110	116	108
Skin disorders	138	106	107	91
Heart failure	100	96	99	90
Esophageal cancer	119	105	107	87
Overall	111	107	111	95

Supplementary Table 2. An Overview of Error Types

Error type	Definition	Example		Consequence
		Source document	Summary	
**Misinterpretation errors**				
Contradiction	Discrepancy between the conclusions drawn from the medical evidence results and the summary	The effect of atypical antipsychotic on psychosis in dementia is negligible	Atypical antipsychotics are effective on psychosis in dementia	Potential for falsehoods
Certainty Illusion	Inconsistency in the degree of certainty between the summary and the source document	There is low-quality confidence that endovascular therapy (ET) plus conventional medical treatment (CMT) compared to CMT alone causes a higher risk of short-term stroke and death	There is moderate-certainty evidence that ET plus CMT compared to CMT alone causes a higher risk of short-term stroke and death	Overly convincing or uncertain summary
**Fabricated errors**				
	No evidence from the source document can be found to support or refute the statement	Two outcomes "help enhance satisfaction and quality of life" are not mentioned in the review	Exercise could help reduce pain and disability and help enhance satisfaction and quality of life for patients with chronic neck pain	Lack of evidence
**Attribute errors**				
Fabricated Attribute	Incorporate an attribute for a specific symptom or outcome that is not referenced in the source document	Population with intracranial artery stenosis (ICAS)	Population with recent symptomatic severe ICAS, where "recent" and "severe" cannot be inferred from the source	Misinterpretation of the review
Omitted Attribute	Neglect an attribute for a specific symptom or outcome	Specify the subtype of dementia	Not specify the subtype of dementia	Overgeneralization of the conclusion
Distorted Attribute	Generate incorrect attribute	Two trials are included in the study	Four trials are included in the study	Misinformation of the study
